# Characterizing monoclonal antibody formulations in arginine glutamate solutions using ^1^H NMR spectroscopy

**DOI:** 10.1080/19420862.2016.1214786

**Published:** 2016-08-11

**Authors:** Priscilla Kheddo, Matthew J. Cliff, Shahid Uddin, Christopher F. van der Walle, Alexander P. Golovanov

**Affiliations:** a Manchester Institute of Biotechnology, University of Manchester, Manchester, UK; b School of Chemistry, University of Manchester, Manchester, UK; c Formulation Sciences, MedImmune Ltd, Granta Park, Cambridge, UK

**Keywords:** Arginine glutamate, mAb formulation, mAb stability, NMR spectroscopy, reversible self-association

## Abstract

Assessing how excipients affect the self-association of monoclonal antibodies (mAbs) requires informative and direct *in situ* measurements for highly concentrated solutions, without sample dilution or perturbation. This study explores the application of solution nuclear magnetic resonance (NMR) spectroscopy for characterization of typical mAb behavior in formulations containing arginine glutamate. The data show that the analysis of signal intensities in 1D ^1^H NMR spectra, when compensated for changes in buffer viscosity, is invaluable for identifying conditions where protein-protein interactions are minimized. NMR-derived molecular translational diffusion rates for concentrated solutions are less useful than transverse relaxation rates as parameters defining optimal formulation. Furthermore, NMR reports on the solution viscosity and mAb aggregation during accelerated stability study assessment, generating data consistent with that acquired by size-exclusion chromatography. The methodology developed here offers NMR spectroscopy as a new tool providing complementary information useful to formulation development of mAbs and other large therapeutic proteins.

## Introduction

Monoclonal antibodies (mAbs) are increasingly being approved as therapeutics, and a substantial number are undergoing evaluation in clinical studies. ^1–3^ However, as proteins, mAbs suffer from instabilities, such as aggregation and self-association, during preparation, formulation and storage, especially at the higher concentrations (>100 mg/ml) often needed to deliver a therapeutic dose as a single injection.^4,5^ Highly concentrated proteins also may form soluble clusters,^6,7^ which may affect the viscosity of solutions,^8^ an important consideration for using such solutions for injections. To minimize the unwanted instabilities, mAbs are formulated in the presence of excipients.^9-19^ New, safe and effective combinations of excipients working synergistically, such as arginine glutamate (Arg·Glu), have been recently described and validated,^20-25^ suggesting that new excipient combinations even within the generally-regarded-as-safe category can significantly improve the storage stability and injectability properties of mAbs.^26^ To assess the suitability of excipients, new orthogonal analytical techniques that are able to report on mAb stability and self-association *in situ* at very high concentrations are needed^27^ because many existing analytical techniques may suffer from observable signals out of scale, thus requiring sample dilution (in turn distorting understanding, e.g., self-association properties). Monitoring such measured physical parameters as a function of excipient type and concentration *in situ*, at the target mAb concentration and temperature (e.g., during accelerated stability studies), would be a direct and undistorted way to choose the best excipients and buffer conditions.

One of the analytical methods currently greatly underused for the formulation characterization of mAbs is solution nuclear magnetic resonance (NMR) spectroscopy. NMR is a very powerful technique capable of observing and monitoring signals from individual groups and types of atoms in a protein molecule, and reporting on the structure and dynamics of proteins in solution.^28-30^ The obvious difficulty of applying solution NMR spectroscopy to mAbs is their large molecular size (ca 145 kDa), which generally leads to broad signals in the spectra and significant signal overlap. Common strategies applied in protein NMR, such as using deuteration or the introduction of isotopic labels, are not generally applicable to full-length native mAbs due to the difficulties with production of such labeled material in the standard expression systems (typically, mammalian cells). The native mAbs solutions that can be characterized have 2 favorable properties: they are generally highly-concentrated, and they allow for higher temperature to be used during the experiments, where the viscosity of water is reduced and molecular tumbling is faster, often leading to NMR spectra of sufficiently good quality. Indeed, recent reports have suggested use of proton NMR and natural-abundance ^1^H-^13^C–correlation spectra to fingerprint mAbs.^31-37^ Because the NMR-observable parameters such as translational and rotational diffusion, transverse relaxation times, deuterium exchange rates and observed signal intensities^28,38,39^ depend strongly on the self-association, aggregation and stability of protein in solution, we explored how such measurable parameters would depend on the concentration and state of a typical industrially relevant IgG1 mAb (identified as “mAb2” in our previous studies^24^) in various solution conditions.

The two aims of the current study were: 1) exploration of the applicability of NMR methodology for typical tasks in protein formulation, and 2) identification of the optimal concentration of Arg·Glu that minimizes mAb self-association and solution viscosity. Here, we used solution NMR spectroscopy to measure a number of experimental parameters for mAb solutions to explore their sensitivity to the changes in the solution environment. The apparent viscosities of solutions derived from NMR measurements were compared with macroscopic solution viscosities measured using the m-VROC viscometer. Accelerated stability studies were also conducted, with NMR detection compared with conventional technique using size-exclusion chromatography (SEC). We suggest a pragmatic approach to interpreting the NMR measurables for optimal formulation development.

## Results

### Using 1D ^1^H NMR spectroscopy to assess mAb stability upon addition of Arg·Glu

Proton NMR signals, which reflect the state of a protein in solution, can be characterized by a number of measurable parameters. Signal integral is generally proportional to the concentration of soluble protein. Protein aggregation increases the rate of transverse relaxation, causing signals to broaden and intensity to decrease. Larger aggregates (e.g., solid sub-micron protein particles) can lead to such a fast signal relaxation that the signals from this sub-species of the sample will not be observable. Therefore, in principle, measuring the intensities of protein signals *vs* different solution environment is expected to report on the aggregation state of protein in solution.

**Figure 1.**
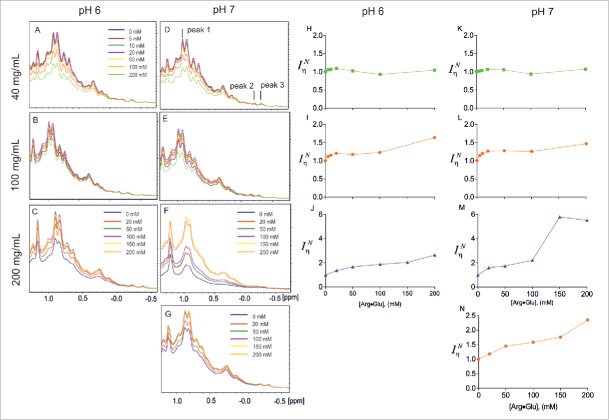
Effect of Arg·Glu addition on NMR signal intensities of mAb2 in different solutions, as labeled. Panels A-G show overlays of selected high-field region of ^1^H NMR spectra of mAb2, with concentrations of components as labeled. In (A)–(F) 10 mM CP buffer was present. Panel (G) includes spectra of 100 mg/ml mAb2 recorded in the absence of any salt apart from Arg·Glu added as indicated. Dependences of viscosity-corrected normalized signal intensities $I_{\eta }^{N}\;$ measured for peak 1 upon increase in Arg·Glu concentration are shown on correspondent right-hand panels (H)–(N). (Color version of this figure is available online)

To assess the effect of solvent conditions on 1D ^1^H NMR spectra of a chosen test mAb (called here mAb2 for consistency with our previous study^24^), we first recorded 1D ^1^H spectra (with identical experimental parameters) for 3 different protein concentrations (40, 100 and 200 mg/ml) at pH 6 and 7, with varying concentrations of Arg·Glu added (between 0 and 200 mM). Respectable spectral quality was achieved at 40°C (see Fig. S1) due to increased molecular tumbling rate at this higher temperature; this temperature is far below the first melting transition temperature for mAb2,^24^ ensuring that the molecule is not significantly destabilized. Results of these experiments are presented on Fig. 1. Several useful observations can be made from looking at the trends (Fig. 1A): the self-association is low when protein is at low concentration (40 mg/ml), and the signal intensities (both at pH 6 and 7,Fig 1A,B) decrease marginally with increased concentrations of Arg·Glu added. This decrease, however, is proportional to the increase in the buffer viscosity (due to Arg·Glu, see below). When the signal intensities are corrected for buffer viscosity ($I_{\eta }^{N}$), they stay fairly flat when mAb2 is at low concentration (Fig 1H, K). For larger concentrations of mAb2 (e.g., 200 mg/ml), the signal behaviors clearly change: despite the increase in buffer viscosity, signal intensities increase with the addition of Arg·Glu (Fig 1C, F). The values of viscosity-corrected normalized signal intensities $I_{\eta }^{N}$ increase even more and grow almost 3-fold and 6-fold at pH 6 (Fig 1J) and pH 7 (Fig 1H), respectively. At the intermediate mAb2 concentration (100 mg/ml), $I_{\eta }^{N}$ show initial faster growth followed by slower growth, with an overall increase of around 1.5-fold when 200 mM Arg·Glu was added (Fig 1I, L). To check if such spectral effects depend on the type and the ionic strength of the base buffer, a control experiment was run for mAb2 dissolved at 100 mg/ml in only de-ionized (Milli-Q) water, where the electrostatic repulsion between the protein molecules is not screened by salt and hence should be at its maximum.^18^ The NMR spectra clearly show that both raw (Fig 1G) and viscosity-corrected normalized $I_{\eta }^{N}\;$ (Fig 1N) signal intensities increase significantly upon addition of Arg·Glu. The increase of signal intensities in NMR spectra recorded under the identical experimental conditions can be unambiguously interpreted as an increase in the population of monomeric or lower-oligomeric protein species and a decrease of concentration-dependent protein self-association^7^ upon the addition of Arg·Glu. Interestingly, addition of Arg·Glu also caused concentration-dependent perturbations of well-resolved high-field mAb2 signals (marked peak 2 and peak 3 on Fig. 1D) from which the disassociation constant *K*_*d*_ for this interaction can be estimated as 90 mM (Fig. S2).

Similar analysis of 1D spectra acquired in the temperature-dependent manner can be used, as well as relative normalized integral parameter $L_{\eta }^{N}$ that we suggest, to assess how excipients or sample conditions affect the melting temperature and amount of soluble mAbs (see Supplemental Information, and Fig. S3). Moreover, by recording the 1D spectra before and after the brief sample exposure to elevated temperature, and using an easily quantifiable NMR-derived parameter that we introduce, a short-term storage stability factor *F*, it is possible to assess the short-term sample stability in different formulations under thermal stress (Supplemental Information, and Fig. S4). We conclude that, as NMR signal intensities are very sensitive to both protein self-association and solution viscosity, finding a formulation that maximizes the signal intensity is expected to coincide with the beneficial formulation leading to stable monomeric mAb solution with minimum overall solution viscosity.

### Accelerated stability studies of mAb2 using NMR and size-exclusion chromatography

**Figure 2.**
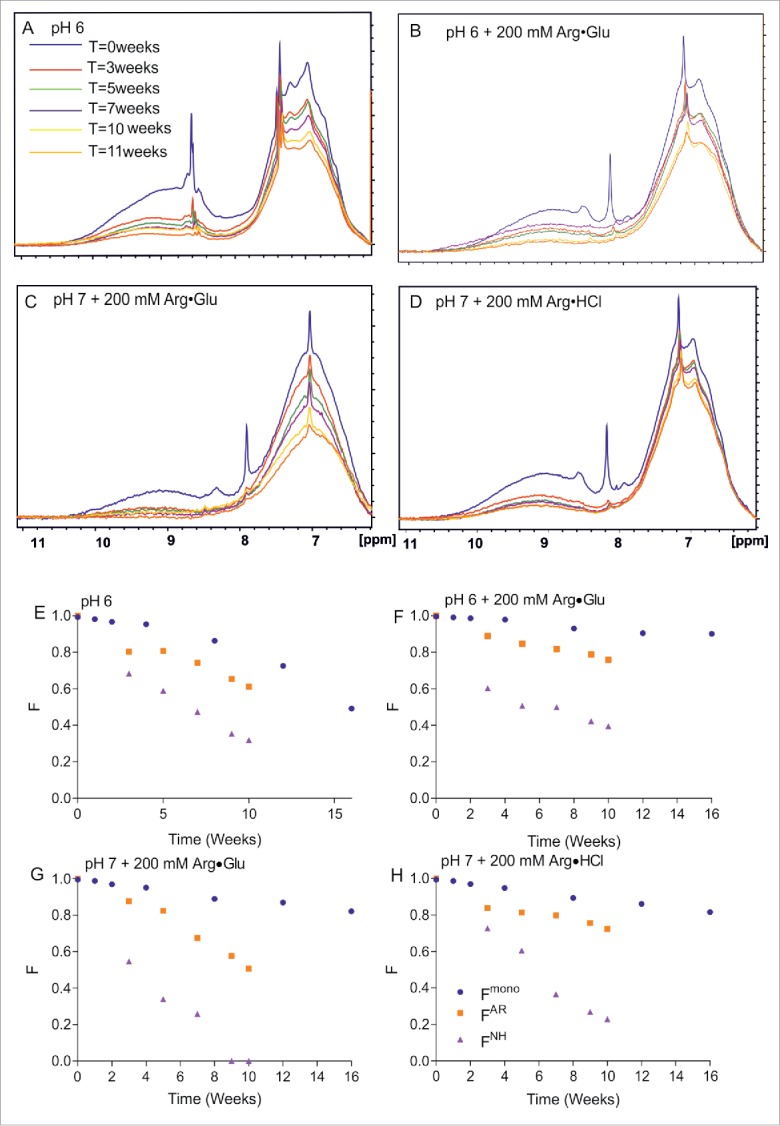
Assessing by NMR and SEC the long-term storage stability of mAb2 at 40°C in selected formulations. The 1D ^1^H NMR spectral overlays (amide and aromatic region) for 4 different formulations of 300 mg/ml mAb2 in 10 mM citrate-phosphate buffer are shown, as a function of time: at pH 6 in the absence of additives (A); in the presence of 200 mM Arg·Glu (B); at pH 7 in the presence of 200 mM Arg·Glu (C); and in the presence of 200 mM Arg·HCl (D). Correspondent panels (E)-(H) show for the same 4 formulations the time-dependence of relative fractions of aromatic (*F*^*AR*^) and amide (*F*^*NH*^) signals remaining in the spectra vs time, reporting on soluble protein loss. Independently, the fraction of monomeric protein *F*^*mono*^ was assessed using SEC and plotted. (Color version of this figure is available online)

Having established that proton NMR signals reflect the amount of monomeric or lower-oligomeric protein remaining in solution, we further explored how NMR can be used to monitor mAb2 physical degradation over time, with concentrated samples (300 mg/ml) stored at 40°C in 4 different formulations. Additionally, to assess the relative exposure of amide groups to the solvent by monitoring the deuterium exchange, NMR samples were formulated in ^2^H_2_O. These long-term storage experiments were also repeated, with the fraction of monomeric protein remaining in solution *F*^*mono*^ assessed by SEC, a traditional method used in industry. The raw spectra for 4 different sample conditions are presented on Fig. 2A–D. The reporter region chosen for monitoring the decrease in peak intensity includes amide region 8–10.5 ppm (region additionally affected by the exchange of protons for deuterons) and region 6–8 ppm, which is mostly populated by the aromatic signals that are not prone to exchange, but with some contribution from exchanging amide signals. These regions were chosen because protein signals here are not obscured by strong signals from the excipients and buffer components used for these formulations. As can be seen from the spectra, with time the signal intensities generally decrease, but the rate of the decrease varies between the 4 chosen formulations. Fig. 2E–H presents the fractions of the initial signal intensities of aromatic signals (*F*^*AR*^) and amide signals (*F*^*NH*^), or of monomer present in solution derived using SEC analysis (*F*^*mono*^), versus time, which all reflect the rate of protein degradation due to sample aggregation and precipitation. It can be seen that monitoring the aromatic signal intensities over time slightly overestimates the rate of apparent sample degradation: signals decrease their intensities with time faster than the monomer is lost in the solution according to SEC analysis.

It should be noted that for the SEC analysis the protein sample needs to be diluted, which is expected to shift the solution equilibrium for reversible self-association toward monomeric species, thus probably overestimating the amount of monomeric protein in the original concentrated solution. This is unlike NMR, which assesses aggregation *in situ*. The difference in the degradation rate also can be explained by additional contribution from deuterium exchange on intensities of amide signals overlapping in the aromatic region. As the rate of deuterium exchange of labile groups (which indirectly reflect on the increase protein dynamics and structure perturbation) is strongly dependent on pH, and is inherently accelerated at higher pH, it is not possible to compare the rates of decay at different pH; however, it is possible to do that at an identical pH. This comparison reveals that adding 200 mM of Arg·Glu significantly increases storage stability at pH 6 (Fig. 2E,F) as reported both by *F*^*mono*^ and *F*^*AR*^, with the rate of deuterium exchange also reduced, as reported by *F*^*NH*^, likely due to more shielding from the solvent in a more stable folded structure. At pH 7, the effect of Arg·Glu was compared with the effect of Arg·HCl. Here, Arg·HCl apparently had more a stabilizing effect than Arg·Glu according to *F*^*AR*^ and *F*^*NH*^, whereas according to *F*^*mono*^ there was not much difference in the long-term stability (Fig. 2G,H). At this point, NMR analysis highlighted the differences in stability between formulations that were not evident from the SEC analysis.

**Figure 3.**
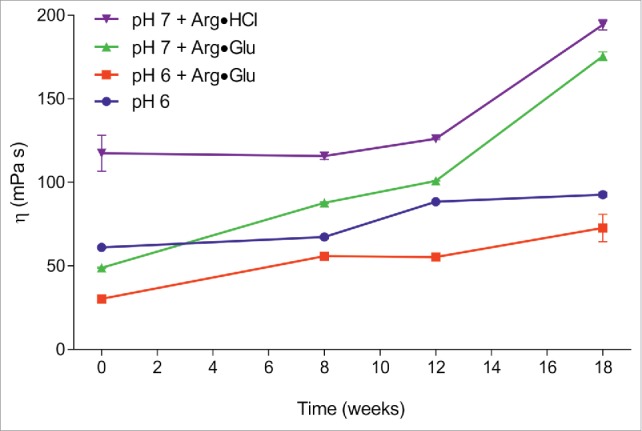
Assessing the macroscopic solution viscosity, using mVROC, during long-term accelerated mAb2 stability studies. mAb2 were formulated at 300 mg/ml in 10 mM citrate-phosphate buffer at pH 6 and further additives, as labeled, and stored at 40°C for prolonged period of time. (Color version of this figure is available online)

In order to understand the reasons for faster decays of *F*^*AR*^ compared to *F*^*mono*^, the solution viscosity needs to be taken into account because its increase (e.g., with time) can also lead to signal decay and additional decrease in measured *F*^*AR*^. To check this hypothesis, the macroscopic viscosities of these 4 formulations were also monitored with time using the m-VROC viscometer (Fig. 3). The measurements reveal that the addition of Arg·Glu leads to much lower mAb2 solution viscosity. Although use of Arg·HCl lead to an apparently stable formulation at pH 7 (Fig. 2H), the viscosity of this formulation was the highest, ∼3–4 times higher than the mAb2 viscosity with Arg·Glu added at pH 6. Interestingly, all formulations tested here showed a tendency to increase in viscosity after prolonged storage. The rate of this increase is not precisely mirrored by the loss of monomer content *F*^*mono*^. The reason for this is unclear, but may reflect the transient nature of reversible self-association of protein oligomers. We suggest that it is this increase in solution viscosity with time that is responsible for the additional decay in *F*^*AR*^, compared with the benchmark *F*^*mono*^ values. As solution viscosity and aggregation are primary considerations in developing mAb formulations, we suggest that monitoring a simple NMR-measurable parameter such as *F*^*AR*^ with time can be a valuable orthogonal criterion for optimizing such formulations. Minimizing the rate of *F*^*AR*^ decay over time should ensure that the maximum amount of soluble un-aggregated protein remains in solution, and solution viscosity is not increased during prolonged storage.

### Assessing viscosity and aggregation state of mAb2 solutions using rheometry and translational self-diffusion measured by SE-PFG NMR spectroscopy

**Figure 4.**
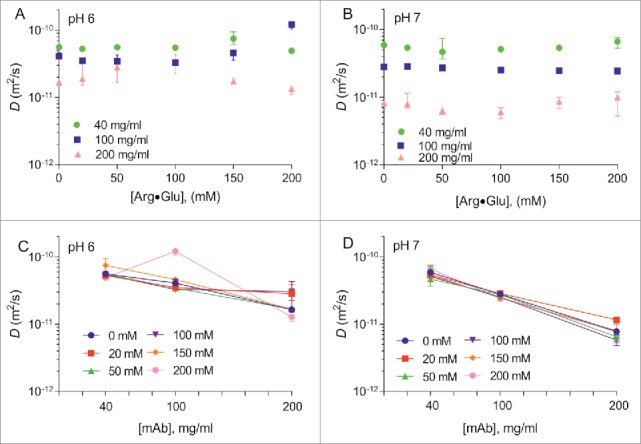
Translational diffusion coefficients *D* of mAb2 measured by DOSY. The values of *D* for mAb2 formulated at pH 6 (A) and pH 7 (B) are shown vs the concentration of Arg·Glu added. The same data is also presented in different coordinates on panels (C) and (D), respectively. (Color version of this figure is available online)

This and previous studies^20,21,23,24^ suggest that addition of Arg·Glu reduces protein aggregation in a concentration-dependent manner. To explore further the effect of Arg·Glu on the apparent protein cluster size and solution viscosity, we employed stimulated echo pulsed-field-gradient (SE-PFG) diffusion-ordered NMR spectroscopy (DOSY)^39-41^ to measure the translational self-diffusion coefficients of both mAb2 and citrate, which served as a small probe molecule in the buffer, at 3 mAb2 concentrations (40, 100 and 200 mg/ml) at pH 6 and 7, in the presence of increasing concentrations of Arg·Glu added up to 200 mM (Fig. S5). Measured diffusion coefficients *D* were plotted as a function of Arg·Glu concentrations (Fig. 4A, B), or for convenience, as a function of mAb2 concentration (Fig. 4C, D). Apart from the observed significant decrease in the values of *D* with increased protein concentration (which was expected due to increased protein crowding, excluded volume effects and protein self-association at higher concentration) the plots of Fig. 4A, B reveal a marginal dependence of diffusion coefficients *D* on concentration of Arg·Glu added. As the translational molecular diffusion rate is dependent on solvent viscosity, and the solvent viscosity inherently increases with the addition of Arg·Glu, this effect needs to be taken into account when interpreting the changes of *D* under different conditions.

**Figure 5.**
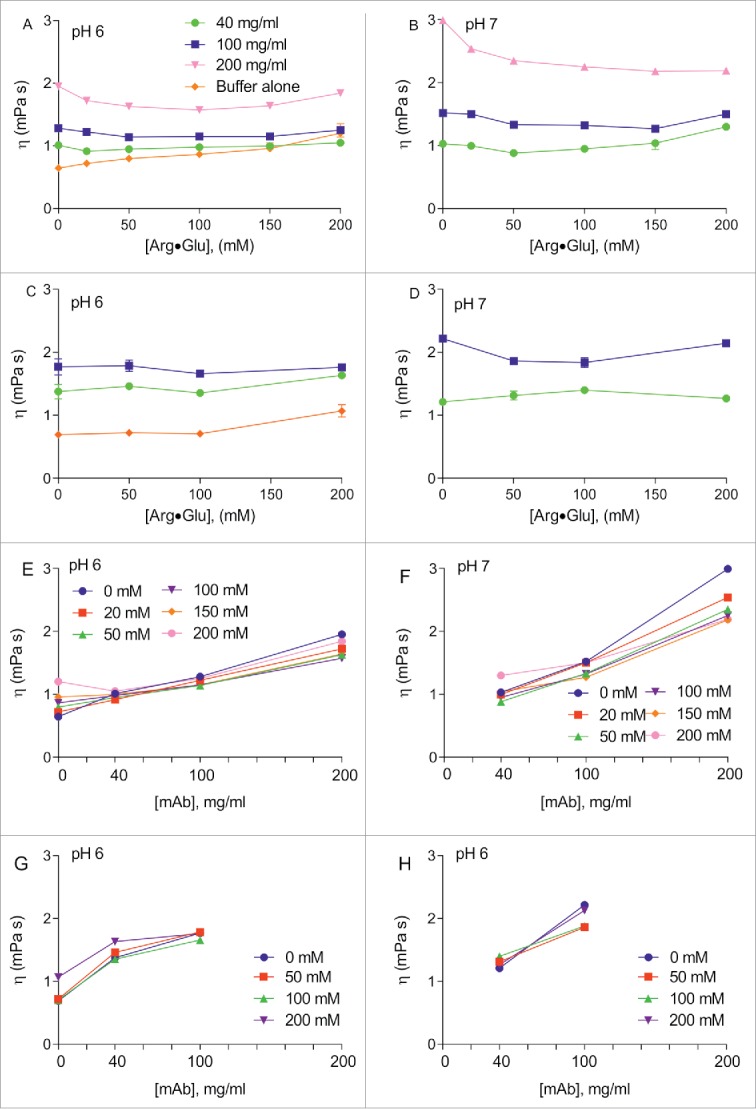
Viscosity of Arg·Glu solutions with and without mAb2 measured by NMR and m-VROC viscometer. Viscosity was measured from NMR-derived diffusion coefficients of citrate ions in solutions at pH 6 (A) and pH 7 (B) in the presence of mAb2 (concentrations as indicated) or buffer alone. Viscosities measured by m-VROC viscometer used the same conditions at pH 6 (C) and pH 7 (D). The NMR data (E, F) and viscometer data (G,H) are re-drawn to highlight the solution viscosity dependence on mAb2 concentration. (Color version of this figure is available online)

The concentration-dependence of the apparent viscosity of the buffer itself, as well as of mAb2 solutions, was measured by following the diffusion of the small probe molecule inherently present in the sample buffer, citric acid (see Materials and Methods). The ‘microscopic’ solution viscosities thus measured by NMR (Fig. 5A, B) were compared to the ‘macroscopic’ solution viscosities measured using the m-VROC viscometer (Fig. 5C, D; due to limited sample availability, the macroscopic viscosity of the 200 mg/ml mAb2 sample was not assessed). The graphs for microscopic and macroscopic viscosities generally follow similar trends, with microscopic viscosities measured for mAb2 solutions by NMR being generally systematically smaller. The macroscopic and microscopic viscosities of the buffer itself upon the addition of Arg·Glu were, however, very similar, showing a steady increase in viscosity (Fig. 5A, C). Despite this increase in the underlying buffer viscosity, the addition of Arg·Glu noticeably decreased the overall viscosity of mAb2 solutions, which was particularly evident at higher protein concentrations (100 and 200 mg/ml), where the solution viscosity was initially very high, with the largest effect observed with 100–150 mM Arg·Glu. This relative decrease in the mAb2 solution viscosity upon addition of Arg·Glu was detected by both NMR and viscometer. We conclude that addition of Arg·Glu to highly concentrated solutions of mAbs can be used not only to increase their stability in storage, but also to reduce viscosity of solutions.

**Figure 6.**
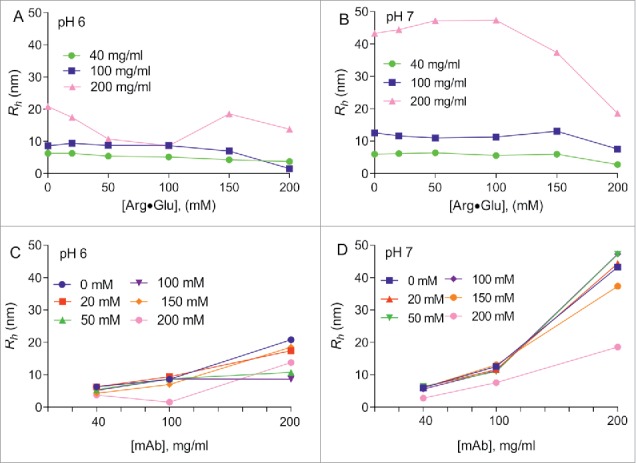
Assessing the changes in the apparent hydrodynamic radius *R*_*h*_ of mAb2 upon addition of Arg·Glu. The values of *R*_*h*_ were assessed vs concentration of Arg·Glu added, using Stokes-Einstein equation for solutions with different concentrations of mAb2 (as labeled) formulated at pH 6 (A) and pH 7 (B). Same dependences are also presented *vs* mAb2 concentrations, with concentrations of Arg·Glu added color-coded, for pH 6 (C) and pH 7 (D). (Color version of this figure is available online)

To explore further the observed effect of Arg·Glu on NMR signal intensities and protein viscosity, we used the Stokes-Einstein Equation 4 (see Materials and Methods) to assess an apparent radius of protein clusters (*R*_*h*_) diffusing in solution, knowing the translational diffusion coefficient *D*, and the measured viscosity of the buffer with Arg·Glu added. Although a crude approximation, the values of *R*_*h*_ may reflect on the apparent changes in the effective cluster size of mAbs forming at higher concentrations, which can be modulated by the addition of Arg·Glu. The results are presented in Fig. 6A, B. At low mAb2 concentration, when self-association of the protein is minimal, the values of *R*_*h*_ appear steady and only marginally decrease upon addition of Arg·Glu, up to the value close to 4 nm expected for a typical monomeric mAb^18^ (Fig. 6A, B). With increased mAb2 concentrations the apparent *R*_*h*_ also increases, but addition of Arg·Glu in the region of 50–100 mM (at pH 6) and 200 mM (at pH 7) caused *R*_*h*_ to drop significantly. For convenience, the same data is presented in different coordinates (Fig. 6C, D), showing more clearly the mAb2-concentration-dependent increase in *R*_*h*_, as well as a partial negation of this effect by addition of Arg·Glu. The reduction in the apparent size of the transient mAb2 clusters upon adding Arg·Glu, revealed here from diffusion measurements, agrees well with the viscosity-reducing effect of Arg·Glu, and matches with the increase in signal intensities in 1D ^1^H spectra described above.

### Measuring the effect of Arg·Glu on proton transverse relaxation rate *R*_*2*_

In addition to translational diffusion, proteins in solution undergo molecular tumbling. The rate of molecular tumbling depends on the size of the cluster, and therefore can report on protein self-association state. The tumbling rate is generally reflected in the value of transverse relaxation rates of the protons *R*_*2*_: the larger the cluster, the faster the rate. However, local polypeptide chain flexibility can reduce the values of *R*_*2*_ for particular signals. Increased relaxation rate *R*_*2*_ makes NMR signals appear broader and decreases signal intensity.

**Figure 7.**
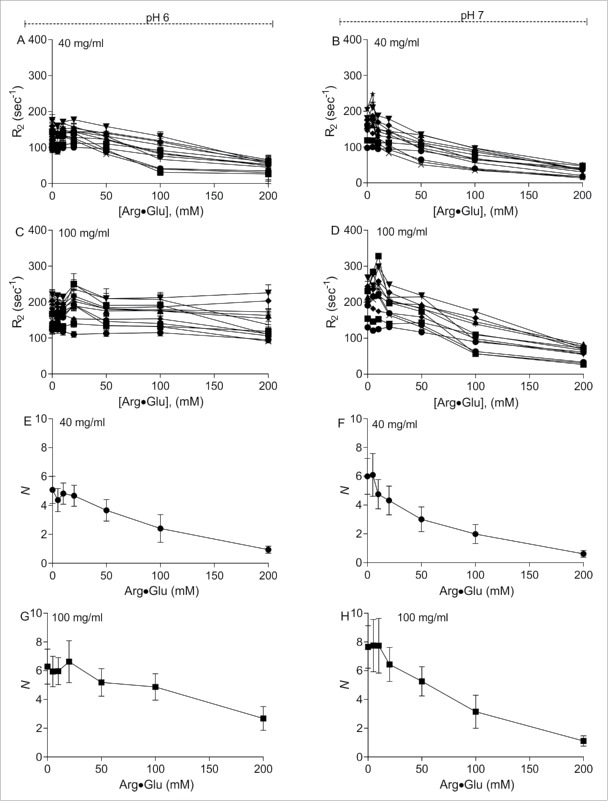
Transverse relaxation rates *R*_*2*_ of mAb2 protons are generally reduced upon addition of Arg·Glu, which can be interpreted as a relative reduction in the effective protein cluster volume. The panels on the left and right correspond to data obtained at pH 6 and pH 7, respectively. Manifolds of the measured dependencies of *R*_*2*_ for a selection of signals of mAb2 formulated at 40 mg/ml (A,B) and 100 mg/ml (C,D) show general downward drift upon addition of increasing concentrations of Arg·Glu. The population average values of *R*_*2*_ were then used to estimate (see Equation 6) the expected relative reduction in the effective aggregation number *N*, for mAb2 formulated at 40 mg/ml (E,F) and 100 mg/ml (G,H). The error bars represent the expected variability of the data in the manifold.

To explore in more detail the effect of Arg·Glu addition on mAb2 signal intensities (Fig. 1), or on the apparent mAb2 radius *R*_*h*_ (Fig. 6), the *R*_*2*_ values were measured for 40 and 100 mg/ml mAb2 solutions at pH 6 and 7, upon addition of increasing concentrations of Arg·Glu. Due to significant overlap between individual proton signals, possible effect of local mobility on relaxation rates of individual protons, and difficulty of tracking the same signals in a titration series, the relaxation data was measured for multiple proton signals in the aliphatic part of the spectra. Thus, the trends in the typical population behavior of *R*_*2*_ values upon addition of Arg·Glu to mAb2 formulated at 40 and 100 mg/ml can be analyzed (Fig. 7A–D). Generally, a significant shift of *R*_*2*_ population was observed toward lower values upon the addition of increasing concentrations of Arg·Glu (Fig. 7A–D), with typical values decreasing around 3-fold upon addition of 200 mM Arg·Glu. Such a dramatic decrease in relaxation rates *R*_*2*_ was unexpected, also taking into account that the inherent increase in buffer viscosity upon an addition of Arg·Glu should slow down molecular tumbling, and contribute toward an increase of *R*_*2*_. It is unlikely that addition of Arg·Glu leads to structure destabilization and increased polypeptide chain flexibility, as mAb2 is equally thermally stable in the presence of Arg·Glu.^24^ To help interpret the significant decrease in transverse relaxation rates *R*_*2*_, we used an empirical observation that, for protein molecules of this size range, the values of *R*_*2*_ are generally proportional to the molecular mass and hence to the effective volume occupied by the molecule or cluster of molecules, as well as to the viscosity of the solution. Using this simple approximation, the averaged relative effective volume (aggregation number) of the mAb2 cluster at each concentration of Arg·Glu was calculated, and these are presented on Fig. 7E–H. The relative values give an estimate of the required change in apparent aggregation number needed to achieve the observed reduction in the measured transverse relaxation rate *R*_*2*_ upon addition of Arg·Glu. We speculate that the estimated typical 6-fold reduction in the apparent aggregation number may reflect the reduction in reversible self-association of mAb2 oligomers, with a concomitant increase in the overall molecular tumbling rate. It should be also noted that the 6-fold reduction in cluster volume can be achieved by only a 1.8-fold decrease in radius, approximating the change in *R*_*h*_ estimated from the translational diffusion measurements (Fig. 6A, B).

## Discussion

It had been long established that the unwanted high viscosity of some mAb formulations originate from reversible self-association that becomes more prominent at higher concentrations.^7,9, 16,27^ Reversible self-association can also be a first step toward formation of irreversible aggregates and particles. Therefore, it is essential to monitor and assess the extent of such self-association, and influence of the sample conditions (pH and excipients), *in situ*, without sample dilution.^27^ Solution NMR spectroscopy is a powerful analytical technique that is used routinely in structural biology, especially for smaller proteins that can be labeled with stable isotopes, ^15^N, ^13^C and ^2^H. This technique is sensitive to even transient interactions between proteins. Ironically, sample optimization to provide the best spectral properties by fine-tuning sample conditions (pH, temperature, additives) to minimize undesired protein aggregation and increase monomeric content, has long been a first standard step in setting up any protein NMR experiment.^42^ Protein NMR typically uses quite high protein concentrations (above mM range), with self-association, increased viscosity and long-term instability of samples all causing problems. The quality, stability and reproducibility of NMR spectra were always used as a criteria for choosing the “best” buffer conditions for a given protein (albeit, usually of small size).

In this study, we explored whether this ‘traditional’ NMR approach can be useful for very large and unlabeled 145 kDa proteins, mAbs, which are normally considered too complex for proton NMR to resolve. We have described a pragmatic approach to NMR data analysis and interpretation, using NMR parameters as criteria for mAb formulation screening, and shown that simply maximizing the signal intensity of the mAb in 1D ^1^H NMR spectra ensures that the size of transient protein clusters, as well as overall solution viscosity, is minimized. The necessary experimental setup, placing mAb samples in NMR tubes in different formulations and running the spectra, followed by the analysis of signal intensities, can be easily automated. Although more high-throughput traditional assays may be beneficial at the early stages of formulation screening, NMR can play a role in later stages where detailed understanding of solution behavior may be beneficial, as well as for orthogonal validation of chosen formulations.

Apart from 1D signal intensities, other NMR measurables, such as translational diffusion and relaxation rates, provide a further insight into mAb behavior in different formulations; analysis of these, however, may require more manual input into the experimental setup and data analysis. We found that, although parameters such as translational diffusion coefficients and transverse relaxation rates may be difficult to interpret in the absolute quantitative sense (as no theory currently adequately addresses the self-interaction of proteins at very high protein concentrations and molecular crowding), these parameters still enable the comparison of different formulation conditions. The unique ability of NMR spectroscopy to provide diverse information about the sample *in situ* and to report on the quantities of monomeric and lower-oligomeric species in solution, as well as their conformational state, is ideally complementary to existing methods such as light scattering and chromatography.

The pragmatic approach taken in this work builds on long-accepted assumptions and simplifications. First, the proton transverse relaxation rate *R*_*2*_ (and hence, signal linewidth) is proportional to the apparent weight-averaged molecular mass.^42,43^ Another implicit assumption, based on the current practice and experience in the protein NMR field, is that observed signal intensities for folded stable proteins in solution are proportional to the concentration of monomeric and lower-oligomeric species, signals from larger aggregates being too broad and unobservable. The decrease in molecular tumbling rate, due to increased viscosity or even transient self-association, will increase transverse relaxation rate and cause signal broadening with concomitant decrease in their intensity. One widely used parameter, protein self-diffusion coefficient, is linked via the Stokes-Einstein equation (4) to the hydrodynamic size of a molecule and solution viscosity. We found that diffusion of small probe molecules, such as citrate present in the buffer, was sensitive to the apparent viscosity of a solution, even when it contained mAbs: correlation was found with the macroscopic solution viscosity. Protein self-diffusion in crowded (concentrated) solutions is well-known to be strongly affected and attenuated by inter-protein collisions during the PGSE diffusion experiment.^40^ In highly-concentrated solutions, this self-diffusion, however, becomes severely limited by the excluded volume and ‘caging’ effect^44^ wherein the diffusion of protein molecules is limited by a high inter-molecular collision rate, although data may still be useful in regard to the relative behavior of comparable solutions.^43^ The translational diffusion may also be a poor reporter of protein association if it is affected by factors such as long-distance electrostatic repulsion.^45^ This may limit the usefulness of protein diffusion coefficient *D* measured at high concentration as a criterion for choosing the ‘best’ formulation condition.

Here, we found that *D* of mAb2 depended strongly on protein concentration, but the effect of Arg·Glu addition on *D* was only marginal, although this excipient addition did have a strong effect judging by the signal intensities and *R*_*2*_ relaxation rates. For interpretation of the changes in *D* (e.g., transforming them into effective changes in radius of hydration *R*_*h*_), the knowledge of solution viscosity is required, but for high-concentration mAbs solutions micro- and macro-viscosity may differ significantly, and adding excipient may further modulate viscosity both directly (usually, increasing micro-viscosity of the buffer) and indirectly (often, decreasing overall mAb solution viscosity). Taking into account that the validity of the Stokes-Einstein equation will be limited for concentrated solutions, deriving the reliable values of *R*_*h*_ from *D* can be open to interpretations and may not be straightforward. The situation with rotational diffusion, which governs *R*_*2*_, is very different: protein can tumble in crowded conditions with a tumbling time τ_c_ dependent on the species size (association state) and transient interactions with neighboring molecules, all of which are sensitive to addition of excipients. Mutual electrostatic steering, which may manifest as transient clusters leading to increased viscosity, would also lead to an increase in *R*_*2*_ rate.^46^ Reduction in such steering by addition of excipients therefore can also be detected.

It should be noted that mAb2 used in this study, which is the same as mAb2 we presented previously,^24^ is an example of an intrinsically stable and soluble antibody. Despite this, at a very high mAb2 concentration the measurable NMR parameters registered quite significant differences as solvent conditions were varied, highlighting the inherent sensitivity of this NMR technique. It can be anticipated that other, less stable mAbs, which require more careful formulation to achieve satisfactory solubility and stability profile, would show even greater variation in NMR measurables. This study also demonstrated that in the NMR experiments it is possible to use significant concentrations (up to 200 mM) of non-deuterated excipients in the samples, without causing noticeable problems with dynamic range, or strong signal overlap. Use of modern NMR spectrometer equipment allows the measurement of relatively weak mAbs signals (with typical concentration 0.26 to 2.0 mM used in this study) on the background of large signals from excipients (e.g., 200 mM), without a necessity to selectively suppress these strong signals. Importantly, the general large dispersion of protein signals allows signals to be picked for analysis that are not obscured by the strong signals from the excipients used. Any baseline distortion introduced can be subtracted from each individual spectrum using the standard spectral processing tools. Moreover, use of existing NMR approaches, for example diffusion-based filtering of signals originating from low-molecular weight excipients, may allow further adaptation of pulse sequences for formulation studies of these large proteins. Introducing the existing tools for automation of sample preparation, spectral acquisition and analysis would allow streamlining of the process and adaptation of this technique for medium-throughput screening environment.

## Materials and methods

### Monoclonal antibody and sample preparation

The monoclonal antibody, mAb2 (IgG1 with MW 145 kDa, pI of 7.9–8.3) was supplied by Medimmune and was identical to mAb2 described in our earlier paper.^24^ Solutions of mAb2, 500 µl each, were prepared in 10 mM citrate-phosphate (CP) buffer at pH 6 and 7 with final concentrations of 40, 100 and 200 mg/mL. To each sample, 5% D_2_O was added for NMR lock. For NMR measurements, parts of these samples (ca 180 µl) were temporarily transferred to 3 mm NMR tubes. To achieve accurately defined addition of Arg·Glu (5–200 mM) without sample dilution, pre-measured aliquots freeze-dried in Eppendorf tubes were successively reconstituted with 500 µl mAb2 solutions. The freeze-dried aliquots of Arg·Glu were prepared from a 0.5 M stock solution containing equimolar mixture of the free amino acids L-Arg (Analytical grade, Sigma–Aldrich) and L-Glu (Analytical grade, Sigma–Aldrich) in MilliQ water, with pH adjusted where necessary. For the long-term stability studies, 4 formulations were prepared by first dialyzing mAb2 in appropriately diluted formulations, freeze-drying the formulations and then reconstituting them in D_2_O in 8-times smaller volume in 180 µl, to achieve the final 300 mg/mL concentration of mAb2 and 10 mM CP buffer in all of them, with additional 200 mM Arg·Glu at pH 6 or pH 7, or 200 mM Arg·HCl at pH 7, or buffer alone at pH 6. Samples were supplemented with 0.01% NaN_3_ to prevent bacterial growth, sealed in 3 mm NMR tubes and stored in a controlled temperature incubator at 40°C for the duration of the study. Final mAb2 concentrations were confirmed based on their absorbance at 280 nm.^24^ For SE-HPLC, mAbs were diluted to 10 mg/mL in the appropriate buffer, with the monomer content quantified as described previously.^24^

### General NMR experiments

All NMR experiments were run on Bruker 800 MHz Avance III spectrometer equipped with 5 mm TCI cryoprobe with temperature control unit, using standard pulse programs and parameters from Bruker library, at 40 °C, unless stated otherwise. Proton 1D spectra were recorded using p3919gp pulse program using 16.0194 ppm spectral width and applying EM window function with typical 10 Hz broadening. Using one 90°-pulse experiment with water presaturation lead to similar changes in signal intensities upon excipient addition, but was not used for quantitative measurements because of more prominent spectral distortions. Spectra were processed and analyzed using Topspin 3.1 and Dynamics Center 2.2.4 (Bruker).

### Analysis of viscosity-corrected signal intensities in 1D ^1^H NMR spectra

To compensate for the increase in buffer viscosity upon addition of Arg·Glu, which slows down molecular tumbling and reduces apparent spectral intensities, the viscosity-corrected normalized signal intensities in NMR spectra $I_{\eta }^{N}$ were calculated as:

(1) $$I_{\eta }^{N}=\frac{I_{\left[RE\right]}^{\;}}{I_{\left[RE=0\right]}^{\;}}\frac{\eta_{\left[RE\right]}}{\eta_{\left[RE=0\right]}}$$

where $I_{\left[RE\right]}^{\;}$ and $I_{\left[RE=0\right]}^{\;}$ are signal intensities and $\eta_{\left[RE\right]}^{\;}$ and $\eta_{\left[RE=0\right]}^{\;}$ are buffer viscosities in the presence and absence of Arg·Glu, respectively. The buffer viscosity values were derived from the diffusion coefficients of citrate ions measured using PFG-NMR spectroscopy (see below). The flat dependencies of $I_{\eta }^{N}\;$ over [Arg·Glu] would show that the concentration of soluble monomeric or lower-oligomeric protein species is not affected by Arg·Glu addition.

### Analysis of temperature dependence of NMR spectra and short-term and long-term thermal stress studies

For these studies, mAb2 at 40 and 100 mg/mL were formulated at pH 6 and 7 with and without 200 mM Arg·Glu, These were subjected to increased temperatures *T* between 40–75 °C, incremented in 5°C steps, with 10 min equilibration after each temperature increase. A pair of 1D NMR spectra (p3919gp pulse program) was then acquired at each temperature with 45 min interval. To assess the dependence of concentration of monomeric or lower-oligomeric soluble species on the temperature *T*, relative increase in viscosity upon addition of 200 mM Arg·Glu, if appropriate, were additionally taken into account. Temperature-dependent normalized integral parameters $L_{\eta }^{N}$ were calculated as:

(2) $$L_{\eta }^{N}\left(T\right)=\frac{L_{\;}^{T}}{L_{\left[RE=0\right]}^{40}}\frac{\eta_{\left[RE\right]}}{\eta_{\left[RE=0\right]}}$$

where $L_{\;}^{T}$ is the signal integral at a particular temperature *T*, $L_{\left[RE=0\right]}^{40}$ is the integral measured at 40 °C in the absence of Arg·Glu, and $\frac{\eta_{\left[RE\right]}}{\eta_{\left[RE=0\right]}}$ is the ratio of the buffer viscosity (with or without 200 mM Arg·Glu, as appropriate) to the viscosity without Arg·Glu. Flat and level dependence of $L_{\eta }^{N}$ over *T* would show that there is no temperature-dependent change in the population of monomeric or lower-oligomeric species.

The fraction of soluble protein *F* preserved in solution after exposure to high temperature for time period *t* were calculated as:

(3) $$F=\frac{I^{t}}{I^{0}}$$

where $I^{0}$ and $I^{t}$ are the intensities of the same signal before and after 45 min exposure at a high temperature. The value F is the measure of short-term sample stability at increased temperature, and shows the fraction loss of monomeric or lower-oligomeric species in solution over an arbitrarily set time period *t* (here, *t* = 45 min). For the long-term stability studies, the samples were stored at 40°C and 1D NMR spectra recorded at the same temperature regularly over 10 weeks. The fraction of soluble monomeric or lower-oligomeric protein preserved in solution after time exposure was calculated using Equation (3) for a number of peaks integrated in the aromatic (*F*^*AR*^) and amide (*F*^*NH*^) regions (7 ppm and 9 ppm respectively), and presented as the fractions of the initial values.

### Measuring diffusion rates by pulsed field gradient (*PFG) NMR spectroscopy*

Changes in the translational diffusion coefficient (*D*) were monitored using SE-PFG (stimulated echo- pulsed-field gradient) with bipolar gradients pulses with water suppression (Bruker's standard pulse program stebpgp1s19). The diffusion time (Δ) and the gradient length (δ) were set to 250 ms and 4.0 ms, respectively. The acquisition time and relaxation delay were 640 ms and 2.0 s, respectively, with a gradient pulse of 45 G/cm. The diffusion spectra were recorded with 32 scans over a spectral width of 16 ppm with 16 linear gradient steps, 10–98% gradient intensity. Each sample was allowed to equilibrate within the NMR spectrometer for 5 minutes after the completion of experimental setup. Translation diffusion coefficients *D* were derived using standard diffusion-ordered spectroscopy (DOSY) analysis offered in Topspin. The errors in *D* were calculated based on the upper and lower error limits for each DOSY peak. The gradients were calibrated to achieve the tabulated values for dioxan diffusion in water,^39^ and then to calibrate the diffusion of citrate ions present in the buffer. Dioxan could not be used as a diffusion probe for buffers containing Arg·Glu due to signal overlap. DOSY experiments allowed to measure diffusion coefficients simultaneously of both probe molecule, citrate, and mAb2, when present, upon addition of Arg·Glu. Thus, measured diffusion coefficient *D* was related to the apparent size of the molecule and apparent viscosity using the Stokes-Einstein equation:

(4) $$\frac{kT}{6\pi R_{h}\eta }$$

where *T* is the absolute temperature, *k* is the Boltzmann constant; *R*_*h*_ is the hydrodynamic radius and η is the viscosity. Diffusion rates of citrate ions in CP buffer at 40°C measured by DOSY without and with Arg·Glu added were used, together with the Equation (4), to determine the values of buffer viscosity in the presence of Arg·Glu, $\eta_{\left[RE\right]}^{\;}$. Parameters were calibrated so that the measured $\eta_{\left[RE=0\right]}^{\;}$ matched the dynamic viscosity of water at 40°C in the absence of Arg·Glu (0.65 mPa s). Using the measured diffusion coefficients *D* of mAb2, and knowing buffer viscosities, the apparent hydrodynamic radius (*R*_*h*_) of mAb2 was calculated using rearranged Equation (4).

### Controlled temperature long-term storage stability studies with HPLC-SEC analysis

Accelerated stability studies were set up at 40°C for 300 mg/mL mAb2 formulated similarly as for NMR long-term stability studies. The final mAb formulations were transferred to 3 ml glass vials and stored for 16 weeks. The samples were tested by SE-HPLC for percentage of mAb2 monomer remaining in solution every week for the first month and then monthly up to 4 months, using the methodology described previously.^24^

### Viscosity measurements

The viscosity of the mAb solutions was determined using the m-VROC viscometer (RheoSense Inc., San Ramon, CA, USA). Solutions of mAb2 at concentrations 40 and 100 mg/ml were measured with a B05-chip at a shear rate of 6000 1/s for 30 sec. Viscosity of 300 mg/ml samples from long-term accelerated stability series was measured using a D05-chip at a shear rate of 5000 1/s for 15 sec. Measurement temperature was set at 40°C and was controlled by an external water bath. Samples were filled in to a 1 ml syringe and triplicate measurements were acquired where possible due to the limitation of sample volumes. Between each measurements the system was washed with 1% tergazyme followed by 1% aquet and then water each with 750 µL/min flow rate for 60 sec (for B05-Chip) or 1000 µL/min for 45 sec (D05-chip).

### Measurement of transverse relaxation rates R_2_

Non-selective proton transverse relaxation rates *R*_*2*_ were measured using a series of standard Carr-Purcell-Meiboom-Gill (CPMG) experiments, with the number of echoes varied (pulse program cpmgpr1d, Bruker). The relaxation delay was 5 sec, and the majority of protein signal decay was occurring between 4 and 66 spin echoes applied (with corresponded times from 2.48 to 40.92 ms). The data was processed using the T1/T2 Relaxation analysis tool in Topspin 3.1 and/or Dynamics Center 2.2.4 (Bruker) and fitted to the mono-exponential decay. To track the changes in the characteristic *R*_*2*_ population behavior upon adding Arg·Glu, 12 prominent proton signals were selected for analysis between −0.5 and 1 ppm and followed throughout the titration. Assuming that *R*_*2*_ of the signals (in the absence of internal motions and chemical exchange) are generally proportional to the rotational correlation time τ_*c*_ ,^42^ which in turn is proportional to the effective spherical volume *V* (or molecular weight) of the protein cluster^39^:

(5) $$R_{2}\propto \tau_{c}=\frac{V\eta }{kT}$$

Further assuming that the lowest value $R_{2}^{m}$, which was observed at the lowest protein concentration 40 mg/ml in the presence of 200 mM of Arg·Glu (with microscopic viscosity η^*m*^), corresponds to the minimum cluster volume *V*^*m*^ (i.e., mAb2 monomer), the apparent aggregation number *N* (i.e, the effective number of mAb2 molecules in a cluster) in all other conditions can be estimated from *R*_*2*_ and known microscopic viscosity η as:

(6) $$N=\frac{V}{V^{m}}=\frac{R_{2}\eta^{m}}{R_{2}^{m}\eta }$$

The value of aggregation number *N* gives an indication of what should be the expected change in the apparent size of the mAb2 cluster to explain the observed decrease in relaxation rate *R*_*2*_ for a rigid molecule.

## Supplementary Material

Supplemental_Data.docx
