# Supplementary material for: Characterizing monoclonal antibody formulations in arginine glutamate solutions using 1H NMR spectroscopy
Source: MAbs. 2016 Aug 11;8(7):1245–58. doi: 10.1080/19420862.2016.1214786 (PMC5058632; doi:10.1080/19420862.2016.1214786)
Supplement: Supplemental_Data.docx [file kmab-08-07-1214786-s001.docx]

**Supplemental information**

**Characterizing monoclonal antibody formulations in arginine glutamate solutions using ^1^H NMR spectroscopy**

Priscilla Kheddo^a,b^, Matthew J. Cliff^a^, Shahid Uddin^c^, Christopher F. van der Walle^c^, Alexander P. Golovanov^a,b^

^a^ Manchester Institute of Biotechnology, University of Manchester, Manchester, M1 7DN, UK; ^b^ Faculty of Life Sciences, University of Manchester, Manchester, M13 9PL, UK; ^c^ Formulation Sciences, MedImmune Ltd, Granta Park, Cambridge, CB21 6GH, UK

**Contact:** Alexander P. Golovanov [a.golovanov@manchester.ac.uk](mailto:a.golovanov@manchester.ac.uk)

**Running title:** Characterizing mAb formulations by NMR

**Supplemental Results**

***Analyzing changes in 1D ^1^H NMR spectra of mAbs upon addition Arg·Glu and increasing the temperature.***

The typical NMR spectrum of mAb2 in the absence of Arg·Glu is shown in Supplemental Fig. S1. In addition to the spectral changes described in the Fig. 1 in the main manuscript, increasing amounts of Arg·Glu also caused concentration-dependent perturbations of well-resolved high-field mAb2 signals (marked peak 2 and peak 3 on Fig. 1D). Assuming that these relative signal shifts were caused by the weak, transient binding of Arg·Glu to mAb2 and fitting their values to a standard one-site binding model (Supplemental Fig. S2), the dissociation constant *K_d_* for this interaction can be estimated as 90 mM.

Having established that the buffer viscosity-corrected normalized NMR signal intensity is a valuable reporter of the state of the protein in solution, the work then explored how normalized signal integral $L_{\eta}^{N}$ is dependent on sample temperature. This established the parameter’s utility in monitoring the combined process of protein melting and aggregation at higher temperatures under different formulation conditions. The ‘best’ formulation condition is expected to maximize both of these parameters. Samples containing 40 and 100 mg/ml of mAb2 at pH 6 and 7, without or with 200 mM Arg·Glu, were heated, and the normalized signal integral $L_{\eta}^{N}$ (see Materials and Methods) was monitored *vs* the temperature. As can be seen on Supplemental Figure S3, at pH 6 when mAb2 concentration is relatively low (40 mg/ml), addition of 200 mM Arg·Glu does not significantly affect protein signal integral or intensity and hence the amount of soluble non-associated protein at lower temperature. However, at 75 $℃$, when the protein starts to unfold,[^24^](#_ENREF_24) the amount of protein remaining in solution is at least two-fold higher than without Arg·Glu (Fig. S3A). At pH 7, which is closer to the mAb2 pI of ~8.1, addition of Arg·Glu reduced the self-association over the whole temperature range, again with ~2.5 times more protein remaining in solution at 75 $℃$ (Fig. S3B). At the higher protein concentration of 100 mg/ml, the stabilizing effect of Arg·Glu is even more evident at both pH 6 and 7, which can be explained by Arg·Glu reducing protein self-association which occurs at this higher protein concentration (Fig. S3C,D). The overall decrease in $L_{\eta}^{N}$ values with increased temperature can be explained by protein loss (due to irreversible aggregation) in the sample. An apparent increase in $L_{\eta}^{N}$ seen for the samples in the absence of Arg·Glu, peaking around 60-65 $℃$ and approaching the values of $L_{\eta}^{N}$ seen in the samples with Arg·Glu (e.g., Fig. S3D), can be explained by higher temperatures dissolving some of the protein clusters,[^7^](#_ENREF_7) and increasing the amount of monomeric and lower-oligomeric species present in solution. In all cases, upon reaching 75 $℃$ all the signal intensities decreased significantly, however the amount of protein remaining in solution in the presence of 200 mM Arg·Glu was always significantly larger, especially at higher protein concentrations. Monitoring the temperature-dependence of viscosity-corrected normalized signal intensities, or normalized signal integrals, therefore can be used to assess how excipients or sample conditions affect the melting temperature and amount of soluble mAbs.

To measure the short-term sample storage stability at elevated temperatures, 1D ^1^H NMR spectra were re-recorded after exposing each sample to a particular temperature stress for 45 min, and calculating the ratio of peak intensities (short-term temperature stability factor *F*) for each temperature (Fig. S4). As the NMR signals from insoluble or larger oligomeric species are generally expected to be too broad and too fast-decaying to be observable, it is reasonable to assume that all observable signals from mAbs originate from the soluble monomeric and lower-oligomeric species. Intensities of two groups of characteristic mAb2 peaks were of particular interest. The signal at 1 ppm (labelled peak 1 on Fig. 1D) is composed of multiple, highly-overlapped methyl and methylene protein signals, and its intensity represents all monomeric and lower-oligomeric protein species, both folded and unfolded, remaining in solution. Two clearly observable up-field shifted methyl signals (peaks 2 and 3 labelled on Fig. 1D) are representative of the well-folded state (<http://www.bmrb.wisc.edu/ref_info/>) of the protein molecule; these signals are lost at higher temperature when the protein denatures, even if the protein stays in solution. Monitoring the short-term stability factors for these signal groups facilitated the distinction between total soluble (both folded and unfolded) protein species which may be present in solution at higher temperature, and soluble natively folded protein. The results shown on Figure S4 reveal that in all conditions tested below 65 $℃$ the protein had a short-term storage stability factor *F* close to 1, meaning that there was no measurable protein loss over 45 min. However, at 70 and 75 $℃$, which is in the melting temperature range, the factor *F* shows a dramatic decrease (Fig. S4A-D). Focusing on these data points (Fig. S4E-H) reveals the pH and mAb2 concentration-dependent nature of these parameters: the net fraction of all soluble protein in the absence of Arg·Glu generally stays the same or decreases as pH is increased from 6 to 7, however in the presence of Arg·Glu the fraction of soluble protein increases significantly, which is particularly well visible at 70 $℃$. The stabilizing effect of Arg·Glu on mAb2 at 40 mg/ml is also visible at 75 ℃: despite protein being largely melted and starting to precipitate, nearly twice the amount remained in solution in the presence of Arg·Glu. These results show that Arg·Glu can stabilize mAb2 and reduce aggregation near its melting temperature point, with pH 7 providing further marginal stability, compared to pH 6. Although in these experiments we chose a fairly short stress period (45 min) at each increased temperature, this period can be increased as required to provide more discriminative power for different formulations at lower temperatures.

**Measuring translational self-diffusion coefficients *D* using DOSY NMR spectroscopy.**

The translational self-diffusion coefficients *D* of both mAb2 and small probe molecule, citrate present in the buffer, at three mAb2 concentrations (40, 100 and 200 mg/ml) at pH 6 and 7, in the presence of increasing concentrations of Arg·Glu added up to 200 mM, was measured using stimulated echo pulsed-field-gradient (SE-PFG) diffusion-ordered NMR spectroscopy (DOSY): ^39-41^ the acquired original DOSY spectra are presented on Figure S5. The behavior of thus measured diffusion coefficients in different formulations is described in the main manuscript.

**Supplemental Figures**


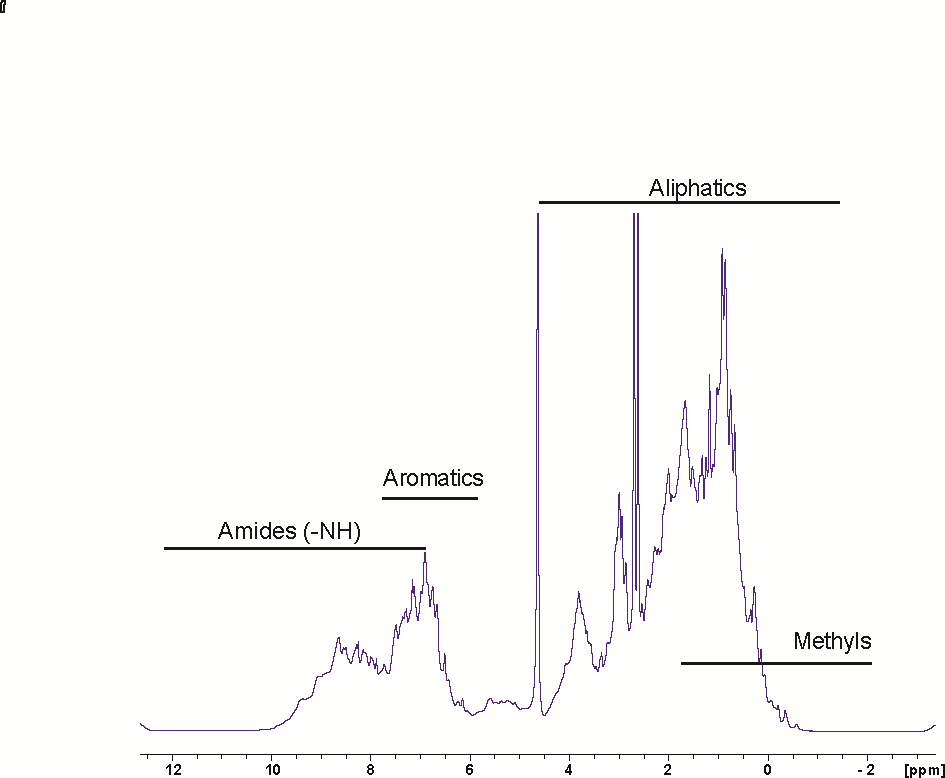


Figure S1. Typical 1D ^1^H NMR spectrum of mAb2. The regions on the spectra reporting on different groups of atoms are annotated accordingly. The strong doublet signal around 2.7 ppm (shown clipped) belongs to the citrate used here as a probe molecule to measure microscopic viscosity.


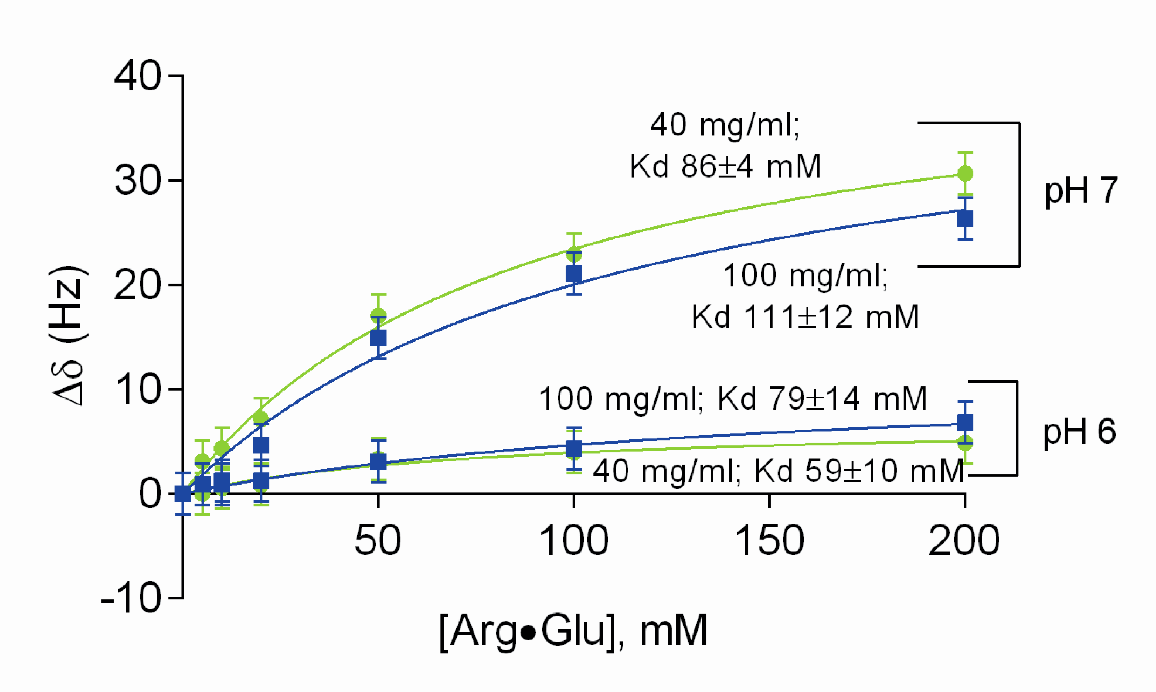


Figure S2. Chemical shift changes between two well-resolved high-field mAb2 signals which are marked peak 2 and peak 3 on Fig. 1D, with increasing Arg·Glu concentrations. The disassociation constant *K_d_* for this interaction was derived by using a one site specific binding equation in GraphPad Prism.


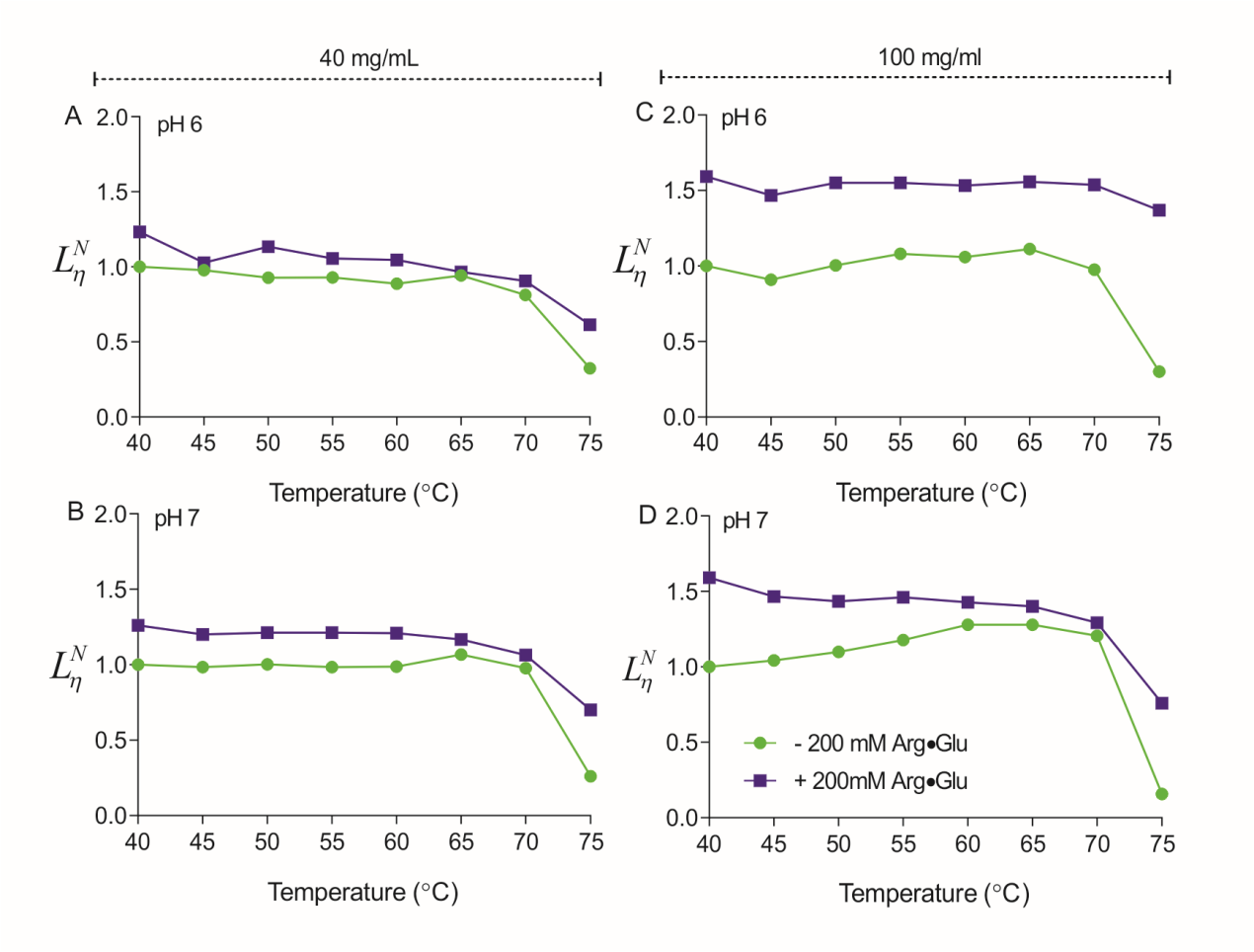


Figure S3. Structural stability of mAb2 at increased temperature with and without 200 mM Arg·Glu. The viscosity-corrected integrals of peaks 2 and 3 (Figure 1D) were additionally normalized to the integral intensities at 40 ℃ in the spectra without Arg·Glu, to yield relative normalized integral parameter $L_{\eta}^{N}$. The measurements were done at pH 6 and 7, and mAb2 formulated at 40 and 100 mg/ml (as labelled).


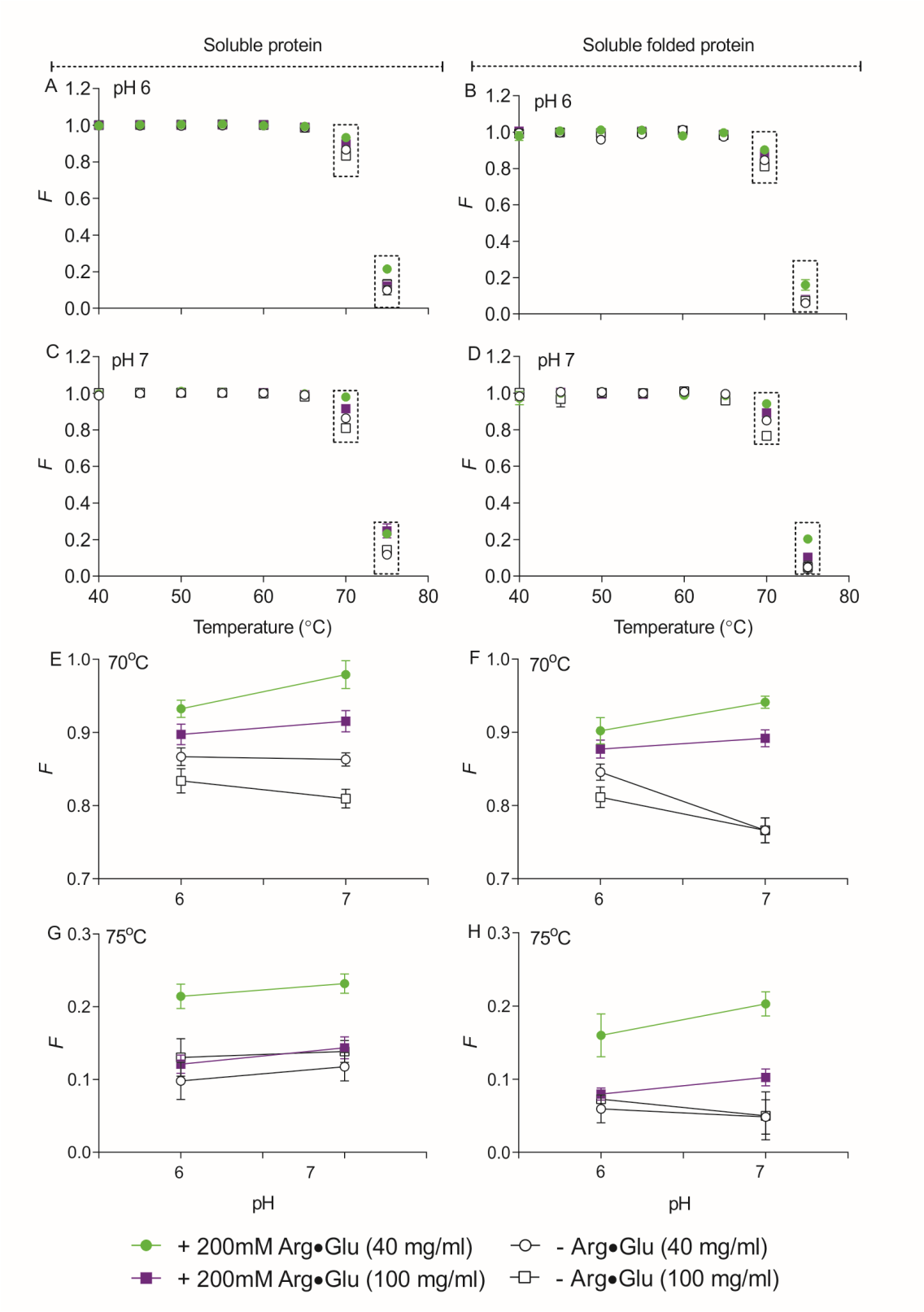


Figure S4. Short-term mAb stability at increased temperatures in the presence (+) and absence (-) of Arg·Glu. Short-term temperature stability factors *F* (calculated as ratio of characteristic signal intensities after/before 45 min temperature stress) are plotted vs temperature (A)-(F), with the highest temperature data points marked in dotted boxes expanded further for clarity and redrawn *vs* pH in panels (E)-(H). Panels on the left-hand side represent total soluble protein present in solution as measured from characteristic peak 1 (cf. Fig. 1D), whereas the right-hand panels represent natively-folded protein present in solution, as measured from characteristic peaks 2 and 3 (cf. Fig. 1D).


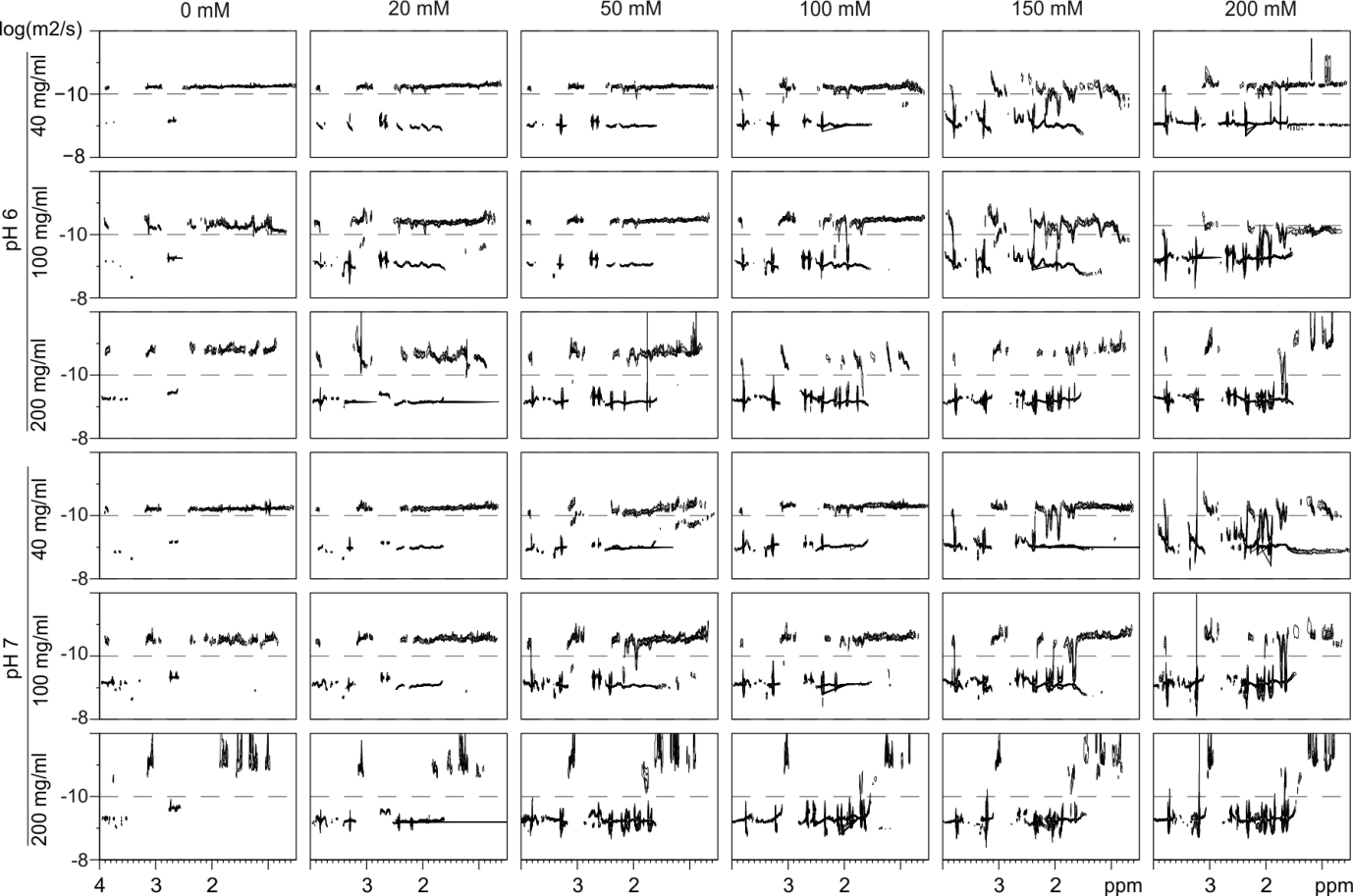


Figure S5. Measuring translational diffusion coefficients of mAbs and citrate ions by DOSY. Collection of DOSY spectra for different mAb2 samples, with increasing concentrations of Arg·Glu added, as labelled. The values of *logD* (in log(m^2^/s) units) are shown on Y axes, and ^1^H chemical shifts on X axis.
